# Transplacental Transmission of Bluetongue Virus 8 in Cattle, UK

**DOI:** 10.3201/eid1512.090788

**Published:** 2009-12

**Authors:** Karin E. Darpel, Carrie A. Batten, Eva Veronesi, Susanna Williamson, Peter Anderson, Mike Dennison, Stuart Clifford, Ciaran Smith, Lucy Philips, Cornelia Bidewell, Katarzyna Bachanek-Bankowska, Anna Sanders, Abid Bin-Tarif, Anthony J. Wilson, Simon Gubbins, Peter P.C. Mertens, Chris A. Oura, Philip S. Mellor

**Affiliations:** Institute for Animal Health, Pirbright, UK (K.E. Darpel, C.A. Batten, E. Veronesi, K. Bachanek-Bankowska, A. Sanders, A. Bin-Tarif, A.J. Wilson, S. Gubbins, P.P.C. Mertens, C.A. Oura, P.S. Mellor); Veterinary Laboratories Agency, Bury St. Edmunds, UK (S. Williamson, C. Bidewell); Animal Health Divisional Office, Bury St. Edmunds (P. Anderson, S. Clifford, C. Smith, L. Philips); Animal Health Divisional Office, Chelmsford, UK (M. Dennison)

**Keywords:** Bluetongue virus, BTV-8, transplacental transmission, pathology, viruses, dispatch

## Abstract

To determine whether transplacental transmission could explain overwintering of bluetongue virus in the United Kingdom, we studied calves born to dams naturally infected during pregnancy in 2007–08. Approximately 33% were infected transplacentally; some had compromised health. In all infected calves, viral load decreased after birth; no evidence of persistent infection was found.

Bluetongue virus (BTV) is generally transmitted between ruminant hosts by *Culicoides* biting midges, and infection may result in the disease called bluetongue. In 2006, a strain of BTV-8 caused the first outbreak of bluetongue in northern Europe (*1*). Although adult *Culicoides* midges are absent from this region during winter for long enough to interrupt normal transmission, BTV-8 survived the winters of 2006–07 and 2007–08.

Several mechanisms have been suggested to explain the overwintering of BTV, one of which is transplacental transmission (*2*). Tissue-attenuated strains of BTV are sometimes capable of crossing the placenta and infecting fetuses in utero (*3*), and transplacental infection has been reported from the field after use of live attenuated vaccines (*4*). However, many wild-type strains of BTV failed to cross the placental barrier when cows were infected during pregnancy (*5*). Additionally, although a few studies have reported experimental transplacental infection with wild-type strains, these studies did not recover infectious virus from live offspring (although many field strains do not grow in tissue culture) and suggested that fetal infection often resulted in deformation, stillbirth, or abortion (*6**,**7*). Collectively, this information led to the assumption that only viruses passaged in tissue culture had the potential to overwinter by transplacental transmission (*8*). However, in 2008, nonlethal transplacental transmission of BTV-8 was detected in Northern Ireland (*9*). To examine the occurrence, rate, and consequences of transplacental BTV-8 transmission in the United Kingdom, we studied calves born to dams naturally infected with BTV-8 during pregnancy.

## The Study

After obtaining owners’ permission, we sampled calves born to previously infected dams during the vector-free period of December 20, 2007 to March 15, 2008. Farmers were also asked to report any births, abortions, or stillbirths from BTV-infected dams outside the vector-free period. Blood samples from live calves were taken as soon as possible after birth (usually within 4 days) and tested by using a real-time reverse transcription–PCR (rRT-PCR) (*10*) and the Pourquier c-ELISA kit (IDEXX, Chalfont St. Peter, UK). When possible, information about the health of the calf was obtained, dams were sampled alongside their calves, and placenta samples were collected. Calves with positive BTV RNA results were resampled at 2–3 week intervals. In total, 61 calves were tested and 21 (including 1 set of twins) had detectable levels of BTV RNA in their blood or organs (Appendix Table). The transplacental transmission rate was 33% (95% confidence interval 22%–47%).

All calves except calf 21 and calf X, each of which had not consumed colostrum before sampling, had antibodies against BTV. Calf 21 was also negative for BTV RNA, but calf X showed the highest viral load in the blood (Appendix Table). Virus isolation in KC cells (*11*) was attempted for all calf blood samples with a cycle threshold (Ct) <29, but virus was isolated from calf X only. Viral RNA load in all calves tested declined over time, and almost all calves were rRT-PCR negative by the end of the study (Table).

**Table:** Bluetongue virus real-time reverse transcription–PCR results from follow-up sampling of calves with initial positive results, United Kingdom, December 20, 2007, to March 15, 2008*

Calf no.	First BTV result, age, d (Ct)	Retest results, age, d (Ct)					Age, d, when PCR negative	Estimated gestation, d†
		Retest 1	Retest 2	Retest 3	Retest 4	Retest 5		
1	15 (25)	28 (26)	44 (26)	58 (28.5)	72 (32.5)	91 (neg)	91	82–219
3	38 (31)	47 (32)	61 (35.5)	81 (neg)	NT	NT	81	106–243
10	79 (32)	106 (33.5)	120 (34)	137 (neg)	158 (neg)	NT	137	140–197
12	81 (28)	108 (30)	122 (31)	139 (34)	160 (neg)	NT	160	142–199
13	4 (33)	31 (36.5)	45 (neg)	62 (neg)	83 (neg)	NT	45	65–122
14	28 (26)	48 (29)	55 (32)	69 (neg)	86 (neg)	107 (neg)	69	154–209
15	70 (32)	97 (neg)	111 (neg)	128 neg)	149 (neg)	NT	97	196–251
20	17 (31)	44 (32.5)	58 (33.5)	75 (neg)	96 (neg	NT	75	78–128
25	27 (29.5)	41 (29)	55 (30.5)	69 (36)	NT	NT	>69‡	145–202
28	1 (23)	26 (25)	35 (26)		NT	NT	>35‡	101–181
29	1 (27)	12 (27.5)	Calf died					45–182
41	47 (28)	61 (29.5)	NT	NT	NT	NT	>61‡	79–126
45	22 (27)	40 (30.5)	61 (34)	NT	NT	NT	>61‡	52–130
47	25 (26.5)	39 (29)	66 (38)	NT	NT	NT	>66‡	52–189
49 (twin with 50)	46 (29)	60 (36)	87 (neg)	NT	NT	NT	87	73–136
50 (twin with 49)	46 (29)	60 (36.5)	87 (neg)	NT	NT	NT	87	73–136
55	21 (25.5)	48 (31.5)		NT	NT	NT	>48‡	34–172

*BTV, bluetongue virus; Ct, cycle threshold; neg, negative; NT, not tested. †Estimated stage of gestation at which transplacental infection may have occurred ‡These calves could not be followed up for farm management reasons or because the project had ended.

When the calves were first sampled, 52 dams were also tested. The RNA load in the calves always exceeded that of their dams, and 7 of the 20 dams giving birth to BTV-positive calves had no detectable viremia.

Of the 21 BTV RNA–positive calves, 5 had compromised health. Calves Y, X, and 33 were born weak and died within hours, days, and weeks after birth, respectively, and calves 13 and 29 exhibited dummy calf syndrome (*12*). All calves except calf 33 were examined postmortem and had negative PCRs for bovine viral diarrhea virus (S.W., pers. comm.). Although calf X died of colisepticemia, this illness probably resulted from the calf’s weakness and inability to consume colostrum. No infectious cause for the early postnatal death of calf Y, other than bluetongue, was identified; pathologic findings for calves 13 and 29 are described elsewhere (S.W. et al., unpub. data). Calf 27, which had negative BTV test results, was born with hypermobility of the fetlock joints, unilateral carpal valgus, and arthrogryposis. All other calves were reported to be healthy.

Time windows for possible in utero infection of each calf were calculated according to the BTV testing history of the dam and the birth date of the calf (Figure). These windows were used to investigate effect of stage of gestation on the probability of transplacental transmission. To account for uncertainty in the date of infection, we used Bayesian methods (Technical Appendix). The probability of transplacental transmission increased with the time of gestation during which the dam became infected (β_1_ 0.033; 95% credibility interval 0.014–0.063).

**Figure:**
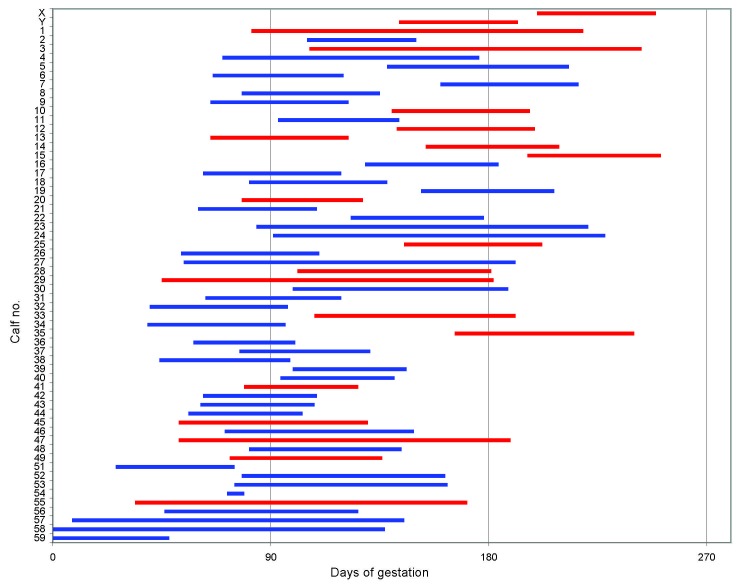
Estimated gestation period at infection of the dam in relation to occurrence of transplacental transmission. Bluetongue virus (BTV) test data for the dams and birth dates of the calves were used to calculate the window of gestation when the dam could have become infected (Technical Appendix, for details). The calculated infection windows are shown in red for BTV-positive calves (transplacental infection did occur) and in blue for BTV-negative calves (transplacental infection did not occur). Because calves were conceived naturally, the exact date of conception is not known, although all were considered to have been born at full term.

## Conclusions

This detailed field study, which combines data on BTV infection in cows with data on transplacentally acquired infection in their offspring, demonstrates that the BTV-8 strain circulating in northern Europe can cross the bovine placenta in a high proportion (33%) of cases and infect calves when dams are infected during pregnancy. A similar study in continental Europe suggested a rate of ≈10% (*13*). However, because the transmission season was longer in some of these countries, many seropositive dams could have been infected before pregnancy, leading to underestimation of the probability of transplacental infection. In our study, we tested only calves from dams infected between August and December 2007 and known to be pregnant at the time of infection. Furthermore, analysis of our data suggests that transplacental transmission is more likely when infection occurs later in gestation; indeed, most of the dams in this study would have been in the second or third gestation trimester when infected (Figure), which may have increased our estimated rate over that found in continental Europe.

Transplacental transmission is of particular concern for policy makers because it may result in the birth of immune-tolerant, persistent carriers, as has happened with bovine viral diarrhea virus (*14*). In our study, all BTV-positive calves other than X and Y were tested after they had received colostrum and, hence, maternal antibodies. The presence of BTV antibodies in calf Y suggests that fetal antibody formed in response to in utero infection, yet calf X had no detectable antibodies against BTV despite strongly positive rRT-PCR results. Calf X was infected late in gestation (Figure), when it should have been capable of mounting its own antibody response (*15*). Antibody-negative PCR-positive calves have been reported elsewhere (*13*). Follow-up testing is needed to assess whether such calves remain persistently infected; however, because calf X died a few days after birth, follow-up testing was not possible.

RNA declined in all retested calves (Table); most were PCR-negative by the end of the study, including dummy calf 13. Therefore, our results do not suggest that transplacental infection with BTV-8 results in subclinical, persistent carriers. Nonetheless, the finding that some calves may be born with deformaties after the virus has cleared may lead to underestimation of the economic effects of BTV; calf 27, which was born with limb deformities to a BTV positive dam, could be such a case.

Live virus has been successfully isolated from only 4 transpacentally infected calves (including calf X described in this study), all of which received either no maternal colostrum or only pooled colostrum (*9*,*13*). Further work is needed to assess whether infectious virus can be isolated from healthy transplacentally infected calves that have colostrum-derived maternal antibodies, because infectious virus needs to be present if transplacental infection is to play a major role in overwintering. In conclusion, future emerging BTV strains should be considered to have the potential for transplacental transmission until investigations show otherwise.

## Supplementary Material

Appendix Table Bluetongue virus testing and results for calves and their dams, United Kingdom*

Technical Appendix Time of Gestation at Infection
