# Supplementary material for: Transplacental Transmission of Bluetongue Virus 8 in Cattle, UK
Source: Emerg Infect Dis. 2009 Dec;15(12):2025–8. doi: 10.3201/eid1512.090788 (PMC3044536; doi:10.3201/eid1512.090788)
Supplement: Appendix Table — Bluetongue virus testing and results for calves and their dams, United Kingdom* [file 09-0788_appT-s1.pdf]

Publisher: CDC; Journal: Emerging Infectious Diseases
Article Type: Dispatch; Volume: 15; Issue: 12; Year: 2009; Article ID: 09-0788
DOI: 10.3201/eid1512.090788; TOC Head: Dispatch

Appendix Table. Bluetongue virus testing and results for calves and their dams, United Kingdom*

Calf details			Dam testing history			Calf test result	
Calf no.	Farm code	Birth date (comment)	Test date	Test result	Reason for sampling	ELISA result	rRT-PCR Ct value for positive results†
X	G	2008 Oct 24 (died 4 d later)	2007 Oct 3	Pos	S	Neg	Ct 18
Y	G	2008 Dec 20 (early postnatal death)	2007 Oct 3	Pos	S	Pos	Pos organs, neg blood
1	A	2008 Feb 19	NA	Pos	P	Pos	Ct 25
2	B	2008 Jan 27	2007 Sep 29	Pos	S	Pos	Neg
3	B	2008 Jan 26	2008 Jan 8	Pos	S	Pos	Ct 31
4	C	2008 Mar 2	2007 Nov 29	Pos	D	Pos	Neg
5	D	2008 Feb 25	2007 Oct 8	Neg	S	Pos	Neg
			2008 Jan 10	Pos	S		
6	D	2008 Mar 6	2007 Oct 8	Pos	S	Pos	Neg
7	E	2007 Dec 3	2007 Oct 11	Pos	S	Pos	Neg
8	E	2008 Feb 23	2007 Oct 11	Pos	S	Pos	Neg
9	E	2008 Mar 7	2007 Oct 11	Pos	S	Pos	Neg
10	E	2007 Dec 23	2007 Oct 11	Pos	S	Pos	Ct 32
11	E	2008 Feb 8	2007 Oct 4	Pos	D	Pos	Neg
12	E	2007 Dec 21	2007 Oct 11	Pos	S	Pos	Ct 28
13	E	2008 Mar 7 (dummy calf)	2007 Oct 11	Pos	S	Pos	Ct 33
14	E	2008 Feb 12	2007 Oct 11	Neg	S	Pos	Ct 26
			2007 Dec 13	Pos	S		
15	E	2008 Jan 1	2007 Oct 11	Neg	S	Pos	Ct 32
			2007 Dec 13	Pos	S		
16	E	2008 Mar 8	2007 Oct 11	Neg	S	Pos	Neg
			2007 Dec 13	Pos	S		
17	E	2008 Mar 10	2007 Oct 11	Pos	S	Pos	Neg
18	E	2008 Feb 20	2007 Oct 11	Pos	S	Pos	Neg
19	E	2008 Feb 14	2007 Oct 11	Neg	S	Pos	Neg
			2007 Dec 13	Pos	S		
20	E	2008 Feb 23	2007 Oct 4	Pos	D	Pos	Ct 31
21	G	2008 Mar 12	2007 Oct 3	Pos	S	Neg	Neg
22	E	2008 Mar 14 (plus placenta)	2007 Oct 11	Neg	S	Pos	Neg, placenta neg
			2007 Dec 13	Pos	S		
23	F	2008 Feb 17	NA	Pos	P	Pos	Neg
24	H	2008 Feb 10	2008 Oct 16	Pos	D	Pos	Neg
25	J	2008 Feb 20	2007 Oct 10	Neg	S	Pos	Ct 29.5
			2007 Dec 14	Pos	S		
26	E	2008 Mar 19 (plus placenta)	2007 Oct 11	Pos	S	Pos	Neg, placenta neg
27	K	2008 Mar 19 (deformed)	2008 Apr 1	Pos	TS	Neg	Neg
28	G	2003 Mar 28	2007 Oct 3	Neg	S	Pos	Ct 23
			2008 Jan 11	Pos	S		
29	F	2008 Mar 27 (dummy calf, died 2008 Apr 8)	2008 Jan 10	Pos	P	Pos	Ct 27
30	L	2008 Mar 21	2007 Sep 24	Neg	S	Pos	Neg
			2008 Jan 11	Pos	S		
31	M	2008 Mar 9	2007 Oct 10	Pos	S	Pos	Neg
32	E	2008 Apr 1	2007 Oct 11	Pos	S	Pos	Neg
33	N	2008 Mar 18 (weak, died ≈4 weeks later)	2007 Sep 30	Neg	S	Pos	Ct 27
			2008 Jan 16	Pos	S		
34	E	2008 Apr 3 (plus placenta)	2007 Oct 11	Pos	S	Pos	Neg, placenta neg
35	P	2008 Jan 29	2007 Oct 9	Neg	S	Pos	Ct 26
			2008 Jan 9	Pos	S		
36	P	2008 Mar 14	2007 Sep 26	Pos	D	Pos	Neg
37	P	2008 Feb 24	2007 Oct 8	Pos	S	Pos	Neg
38	P	2008 Mar 28	2007 Oct 8	Pos	S	Pos	Neg
39	Q	2008 Feb 2	2007 Oct 1	Pos	S	Pos	Neg
40	Q	2008 Feb 7	2007 Oct 1	Pos	S	Pos	Neg
41	Q	2008 Feb 22	2007 Oct 1	Pos	S	Pos	Ct 28
42	Q	2008 Mar 10	2007 Oct 1	Pos	S	Pos	Neg
43	Q	2008 Mar 11	2007 Oct 1	Pos	S	Pos	Neg
44	Q	2008 Mar 16	2007 Oct 1	Pos	S	Pos	Neg
45	K	2008 Mar 20	2007 Nov 1	Pos	D	Pos	Ct 27; placenta Ct

		(plus placenta, no placentome)					29.5
46	K	2008 Mar 1	2007 Nov 1	Pos	D	Pos	Neg
47	R	2008 Mar 20	2008 Apr 14	Pos	TS	Pos	Ct 26.5
48	R	2008 Feb 20	2007 Oct 17	Pos	D	Pos	Neg
49	R	2008 Feb 28 (twins)	2007 Oct 17	Pos	D	Pos	Ct 29
50							
51	G	2008 Apr 15	2007 Oct 3	Pos	S	Pos	Neg
52	B	2008 Apr 16	2007 Sep 29	Neg	S	Pos	Neg
			2008 Jan 8	Pos	S		
53	S	2008 Apr 15 (plus placenta)	2007 Sep 25	Neg	S	Pos	Neg, placenta neg
			2008 Jan 9	Pos	S		
54	S	2008 Apr 11	2007 Sep 25	Pos	S	Pos	Neg
55	R	2008 Apr 7	2008 Apr 28	Pos	TS	Pos	Ct 25.5
56	T	2008 Mar 26	2007 Nov 3	Pos	D	Pos	Neg
57	U	2008 May 3	2008 Feb 20	Pos	P	Pos	Neg
58	V	2008 May 16	NA	Pos	P	Pos	Neg
59	W	2008 May 22	2007 Oct 2	Pos	S	Inconclusive	Neg

*rRT-PCR, real-time reverse transcription–PCR; Ct, cycle threshold; pos, positive; neg, negative; S, surveillance; P, premovement (premovement tests were sometimes conducted by ELISA only); D, diagnostic (disease reported); TS, transplacental study only (dam had not been tested before study, but farmer suspected infection).

†Low Ct value indicates a high level of viral RNA and vice versa. Samples were run in duplicate, and averages are given. If no Ct was detected, the sample was classified as negative.
