# Supplementary material for: Transplacental Transmission of Bluetongue Virus 8 in Cattle, UK
Source: Emerg Infect Dis. 2009 Dec;15(12):2025–8. doi: 10.3201/eid1512.090788 (PMC3044536; doi:10.3201/eid1512.090788)
Supplement: Technical Appendix — Time of Gestation at Infection [file 09-0788_Techapp-s2.pdf]

Transplacental Transmission of Bluetongue Virus 8 in Cattle, UK

Technical Appendix

Time of Gestation at Infection

All calves were conceived through natural breeding, therefore, the exact date of insemination was unknown. However, all calves were considered by the farmers to have been born at full term. BTV testing history of the dam combined with the birth date of the calf were used to calculate the stage of gestation at which infection was likely to have occurred. Assuming a gestation period of 280 days, the earliest and latest times during gestation at which the dam could have been affected were estimated as

$$\begin{aligned} t_i^{(L)} &= 280 - (B_i - I_i^{(L)}), \\ t_i^{(U)} &= 280 - (B_i - I_i^{(U)}), \end{aligned} \quad (1)$$

where B_i is the date of birth of calf i, $I_i^{(L)}$ is the earliest date of infection (either 2 days before the last negative PCR result, if available (see Table 1 and Figure 1 in main paper) or 05 August 2007, the most likely date for the introduction of BTV to Great Britain (*1*) and $I_i^{(U)}$ is the latest date of infection (the earlier of ten days before the first positive ELISA result (table 1 and figure in main paper) or 20 December 2007, the date on which the "vector-free" period was declared (*2*).

Transplacental Infection in Relation to Time of Gestation and Pregnancy

The probability of transplacental transmission from dam to calf for the *i*th calf-dam pair is given by

$$\log\left(\frac{p(t_i)}{1 - p(t_i)}\right) = \beta_0 + \sum_{j=1}^{m} \beta_j t_i^j, \ (2)$$

where t_i is the stage of gestation at which the *i*th dam was infected. A Bayesian approach assuming non-informative (diffuse Normal) priors was used to estimate the parameters (the β_is). The model was implemented using WinBUGS (*3*) to generate posterior densities for

each parameter that allow for the uncertainty in the time of infection for the dam. In this case, the time of infection was sampled from a uniform distribution

$$t_i \sim \text{uniform}\left(t_i^{(L)}, t_i^{(U)}\right)$$

where $t_i^{(L)}$ and $t_i^{(U)}$ are the earliest and latest times during gestation at which the dam could have been affected, respectively (defined in equation (*1*)). The final model for the probability of transplacental transmission was constructed starting from a linear function (m = 1) in equation (*1*) and sequentially adding higher-order terms (m = 2,3,...) until there was no improvement in model fit, as judged by the Deviance Information Criterion (DIC) (*4*). Multiple chains were run to check convergence and, in each case, estimates were based on 50,000 iterations of the chain, with the preceding 10,000 iterations discarded.

The probability of transplacental transmission was adequately described by a linear model (DIC = 64.32); adding a quadratic term did not improve model fit (DIC = 64.44). The final model indicated that the probability of transplacental transmission increased as the time of gestation at which the dam became infected increased (β_1 = 0.033; 95% credibility interval: 0.014–0.063).

References

1. Gloster J, Burgin L, Witham C, Athanassiadou M, Mellor PS. Bluetongue in the United Kingdom and northern Europe in 2007 and key issues for 2008. Vet Rec. 2008;162:298–302. PubMed

2. Department for Environment, Food and Rural Affairs. Bluetongue update: vector-free period confirmed and movement restrictions relaxed. Defra News Release 21/12/2007. http://ndscoigovuk/content/detailasp?ReleaseID=340775&NewsAreaID=2

3. Lunn DJ, Thomas A, Best N, Spiegelhalter D. WinBUGS - a Bayseian modelling framework: concepts, structure and extensibility. Stat Comput. 2000;10:325–37. DOI: 10.1023/A:1008929526011

4. Spiegelhater DJ, Best NG, Carlin BP, van der Linde A. Bayesian measures of model complexity and fit (with discussion). Journal of the Royal Statistical Society B. 2002;64:583–639. DOI: 10.1111/1467-9868.00353
